# Parasites and lameness in domestic animals

**DOI:** 10.1007/s11259-025-10754-4

**Published:** 2025-04-30

**Authors:** Ibrahim Akin, Ozge Ozcan, Yalcin Alper Ozturan

**Affiliations:** Department of Surgery, Faculty of Veterinary Medicine, Aydin Adnan Menderes University, 09100 Aydin, Turkey

**Keywords:** Cat, Dog, Equidae, Lameness, Parasites, Ruminant

## Abstract

Lameness is a significant welfare issue in domestic animals, and it may also result in productivity losses in farm animals. While traumatic injuries are the primary cause of lameness in animals, parasitic infections may be a potential factor in cases where the underlying cause of lameness remains unclear. Parasites may cause lameness in animals by inflicting extensive pathological damage to various organs and tissues, inducing severe anemia, producing endo- and exotoxins that act on the host, and more. However, the mechanisms by which many parasites induce lameness remain unknown. This review summarizes the literature on parasitic-induced lameness, which is classified as direct or indirect. Direct lameness occurs when parasites damage tissues such as muscles, bones, joints, tendons, and nerves, leading to a loss of function in these structures and subsequent lameness. Neurotoxins produced by some parasites may also cause coordination disorders, paralysis, and lameness in animals. Indirect lameness is caused by Égaré parasites—misplaced parasites that reside in tissues unrelated to their typical target location. These parasites may be found in the central nervous system, muscles, nerves, tendons, interdigital region, or femoral artery. Some endoparasites may cause tissue damage during migration, while others may induce lameness by affecting the circulatory system through blood parasites.

## Introduction

Lameness is a significant welfare issue in animals that may cause pain and discomfort, reduce mobility, and negatively impact productivity (Bruijnis et al. 2013; Herrero and Thornton 2013; Koeck et al. 2014; Liang et al. 2017; Akin and Akin 2018). Lameness in domestic animals can result from various causes, including inflammatory processes (e.g., bacterial, viral, or other infections) and metabolic disorders (e.g., arthritis, laminitis) that lead to structural or functional impairment of the musculoskeletal system. In some cases, parasitic infections may also contribute to lameness, particularly when the underlying cause remains unclear and is not detected through routine clinical practices (Leonard and Tillson 2001; Rochat 2005). Parasites may cause lameness in animals by inflicting extensive damage to various organs and tissues, inducing severe anemia, and producing endotoxins and exotoxins that effects the host (Poitout et al. 2005; Tarello 2005; Gómez et al. 2010; Moroni et al. 2012). However, the mechanisms by which many parasites induce lameness remains poorly understood. Endoparasites may cause tissue damage during migration, while blood parasites may affect the circulatory system and cause lameness (Poitout et al. 2005; Tarello 2005; Gómez et al. 2010; Moroni et al. 2012).

Parasitic-induced lameness may be classified as either direct or indirect. Direct lameness results from the damage that parasites inflict on tissues such as muscles, bones, joints, tendons, and nerves, leading to a loss of function in these structures and subsequent lameness (Morelli et al. 2021). Neurotoxins produced by some parasites may also cause coordination disorders, paralysis, and lameness in animals (Poitout et al. 2005; Tarello 2005; Gómez et al. 2010; Moroni et al. 2012). Conversely, indirect lameness is caused by parasites found in tissues unrelated to their usual location. They may be present in the central nervous system, muscles, nerves, tendons, interdigital region, or femoral artery (Morelli et al. 2021). Effective parasite control strategies are essential for preventing and managing parasitic-induced lameness in domestic and farm animals.

This review aims to underscore the need for continued research into the diverse clinical manifestations of parasitic infections—particularly lameness—and the underlying mechanisms involved. A deeper understanding of these interactions could support the development of more effective parasitic control strategies, especially in cases where lameness may indicate a more serious systemic condition. In this narrative review, we synthesize current knowledge on parasitic causes of lameness in domestic animals, specifically focusing on dogs and cats, ruminants, equids, and pigs. A comprehensive literature search was conducted using relevant key terms such as ‘lameness,’ ‘parasitic infections,’ and the parasites discussed in this article, along with terms like ‘domestic animals,’ ‘ruminant,’ ‘dog,’ ‘cat,’ ‘horse,’ ‘equid,’ ‘sheep,’ ‘cattle,’ ‘cow,’ ‘goat,’ and ‘pig’ across various scientific databases (PubMed, Google Scholar, CABI, and Scopus). Studies were selected based on their relevance to parasitic infections associated with lameness, clinical presentation, and management approaches. Inclusion criteria focused on studies addressing parasitic causes of lameness, while exclusion criteria excluded non-parasitic causes and studies lacking sufficient clinical data. The selected studies were reviewed thematically, offering a comprehensive overview of the topic. As a narrative review, the study selection process was broad, allowing for an extensive exploration of the subject without adhering to the rigid protocols required for systematic reviews. This approach ensures a well-rounded understanding of the relationship between parasitic infections and lameness in farm animals, emphasizing the importance of this issue in veterinary practice. The review presents parasitic-induced lameness in cats and dogs, ruminants, and equids under separate headings.

## Parasites and lameness in dogs and cats

**Fig. 1:**
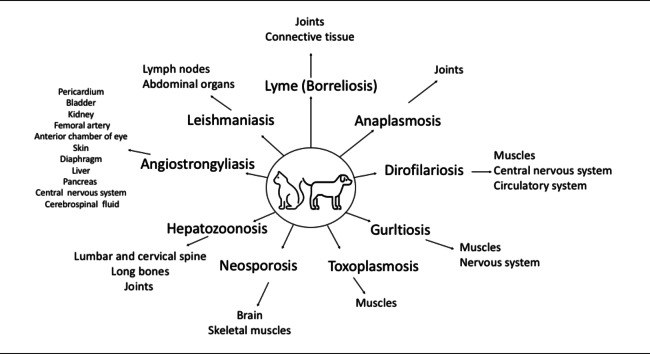
Parasitic diseases and affected structures and tissues in dogs and cats

Parasitic diseases and the affected structures and tissues in dogs and cats presented in Fig. 1. External parasites include lice (*Phthiraptera*), fleas (*Siphonaptera*), ticks (*Ixodida*,* Amblyomma*,* Rhipicephalus*,* Haemophysalis*,* Dermacentor*), and mange mites (*Sarcoptes scabiei*) and may cause problems for domestic animals (Hopla et al. 1994). Among these, ticks have been reported to be the most common cause of lameness in animals in various literatures (Poitout et al. 2005; Rizzoli et al. 2011; Halperin 2015). Ticks can contribute to lameness by transmitting disease agents such as *Borrelia burgdorferi* and *Anaplasma phagocytophilum* to the host. Additionally, certain tick species have been implicated in causing lameness through the secretion of neurotoxins. The risk of infection after a single tick bite is low, even in endemic areas (Costello et al. 1989). However, it has been reported that ticks may acquire and transmit multiple pathogens over the course of their life (Levin and Fish 2000). The transmission of pathogenic agents (bacterial, viral, parasitic, etc.) from ticks to hosts may lead to lameness, with the severity depending on the type of infection, the duration of tick attachment, and the health or immune status of the host. Various parasites associated with lameness in dogs and cats, including those transmitted by ticks, are summarized in Table 1. Further information on parasitic diseases associated with lameness in dogs and cats is presented under the headings below.

**Table 1:** Parasitic diseases associated with lameness in dogs and cats: causative agents, intermediate hosts, tissue involvement, and diagnostic methods

Diseases	Causative		Tissue	Diagnosis method
	Intermediate host(s)	Parasite(s)		
Lyme (Borreliosis)	*Borrelia burgdorferi*	*Ixodes ricinus*	Joint Connective Tissues	PCR
Anaplasmosis	*Anaplasma phagocytophilum*	*Ixodidae* spp.	Joint	Synovial Fluid Analysis
Dirofilariosis	Aedes (Ae.), Anopheles (An.), Culex (Cx.) and Ochlerotatus (Oc)	*Dirofilaria immitis*	Muscles, CNS, Circulatory System	Blood Sample Examination for Microfilaria
Gurltiosis	Gastropods	*Gurltia paralysans*	Muscles, CNS	Necropsy
Toxoplasmosis	Mouse	*Toxoplasma gondii*	Muscles	Blood Sample, BAL, CSF, Peritoneal Fluid, Tear and Tissue Samples
Neosporosis	Contaminated waste and animal feces	*Neospora caninum*	Brain, Skeletal Muscles	Microscopic Examination, ABC, ELISA
Hepatozoonosis	*Rhipicephalus sanguineus*	*Hepatazoon americanum*	Lumbar and Cervical Spines, Long Bones, Joints	Serological Tests, ELISA
Angiostrongyliasis	Gastropods	*Angiostrongylus cantonensis*	Pericardium, Bladder, Kidney, Femoral Artery, Eye, Skin, Diaphragm, Liver, Pancreas, CNS, CSF	Radiography, CT, Cytology, Serological Tests, ELISA
Leishmaniasis	Phlebotomus and Lutzomyia	*Leishmania infantum* *Leishmania chagasi* *Leishmania tropica*	Lymph Nodes, Abdominal Organs	Microscopic Examination, Tissue Cultivation Delayed Hypersensitivity Test Serological Tests Molecular Diagnostics

*PCR* Polymerase chain reaction, *CNS* Central nervous system, *BAL* Bronchoalveolar lavage, *CSF* Cerebrospinal fluid, *ABC* Avidin-biotin-peroxidase complex, *CT* Computed tomography

### Lyme (Borreliosis)

Lyme disease, also known as Borreliosis, is one of the most commonly diagnosed zoonotic vector-borne diseases worldwide. The primary cause of the disease is the *Borrelia burgdorferi* bacteria, and the *Ixodes* genus ticks play an essential role in its transmission (Burgdorfer 1989). It has been reported in Turkiye that *Ixodes ricinus* acts as a vector for *B. burgdorferi* transmission (Güner et al. 2003). The acute phase of *Borrelia* infection is characterized by weakness, lameness, arthritis, fever, and lymph node enlargement. In chronic cases, arthritis may occur after treatment. The clinical symptoms of the disease may appear months after contact with the tick. Intermittent lameness and mono-oligoarthritis (involvement of a single joint or multiple joints) may occur 2–5 months after contact with the tick (Rizzoli et al. 2011). In the final stage of *Borrelia* spp. infection, spirochetes may enter the narrow joint spaces and cause arthritis in approximately 10% of patients (Halperin 2015). The spread of *Borrelia* spirochetes in the skin, joint, and connective tissues, along with local inflammatory reactions, may lead to clinical symptoms such as pain, lameness, and swelling. Synovial fluid polymerase chain reaction (PCR) testing is expected to be positive for the diagnosis of the disease (Miller and Aucott 2021). The relationship between *Borrelia* infection and its clinical signs in cats remains unclear (Shaw et al. 2005) and warrants further research.

### Anaplasmosis

Anaplasmosis is a disease characterized by fever, which is transmitted by ticks of the Ixodidae family, infected with *Anaplasma phagocytophilum*. Infected dogs may show no symptoms or exhibit symptoms such as lameness, anorexia, lethargy, vomiting, and diarrhea (Poitout et al. 2005; Barutzki et al. 2006). In infected dogs, lethargy, anorexia, fever, and lameness have been reported to occur due to lymphopenia, thrombocytopenia, and increased serum alkaline phosphatase (Poitout et al. 2005; Barutzki et al. 2006). Studies have reported that lameness is a prominent sign in orthopedic examinations of dogs infected with *Anaplasma phagocytophilum* (Poitout et al. 2005; Barutzki et al. 2006; Kohn et al. 2008). In a study conducted in Germany to determine the seroprevalence of *Anaplasma phagocytophilum* in dogs (Barutzki et al. 2006), serum samples from 1124 dogs suspected of having anaplasmosis were tested, and antibodies were detected in 563 dogs (50.1%). Clinical signs were evaluated in a subgroup of 26 seropositive dogs, of which 13 (50%) exhibited lameness. In another study conducted over 26 months in Berlin (Kohn et al. 2008), lameness was observed in 2 out of 18 naturally infected dogs (11.1%). One of the lame dogs exhibited joint swelling, and synovial fluid analysis revealed cloudy fluid with significantly reduced viscosity. The variation observed between these studies may be attributed to differences in study design, case selection, and disease stage. While Barutzki et al. (2006) included a broader subset of seropositive dogs—potentially exhibiting a range of clinical presentations—Kohn et al. (2008) focused specifically on dogs with naturally occurring symptomatic infections. These methodological differences, along with individual host responses and the presence or absence of joint involvement, likely contribute to the reported disparities in the frequency of lameness. In cats a study conducted in Italy on 15 cats (Tarello 2005) found that lameness, hyperesthesia, anorexia, lethargy, muscle and joint pain, neck stiffness, coordination disorders, and hyperglobulinemia were among the symptoms of *Anaplasma phagocytophilum* infection. In a study conducted in Poland (Adaszek et al. 2013), swelling and pain were reported in the joints of three cats infected with *Anaplasma*.

### Dirofilariosis

Endoparasites are parasites that live inside the host’s body, and their development may involve migration through tissues, which may cause damage to the host. For example, *Dirofilaria immitis* may obstruct tissues or organs, while *Gurltia paralysans* may settle in muscles, the central nervous system, or the circulatory system, causing functional impairment and lameness. Additionally, some endoparasites may settle in tissues where they shouldn’t, leading to lameness indirectly. Heartworm disease, caused by *Dirofilaria immitis* in carnivores, is known to cause lameness due to abnormal migration and has been reported to cause circulatory, respiratory, and systemic diseases in both carnivores and humans (Meral et al. 2007). Several studies have documented the abnormal migration of adult *Dirofilaria* to various tissues (Hodges and Rishniw 2008; Choi et al. 2012; Oldach et al. 2018).

### Gurltiosis

Some parasites may cause damage to the nervous tissue by passing through the brain or spinal parenchyma during their migration. Depending on the organism they pass through, it has been reported that they may cause hemorrhage and embolus, vasospasm, acute infarction, space-occupying lesions, and local inflammatory reactions (Williams et al. 1998). *Gurltia paralysans*, a metastrongyloid parasite, causes chronic meningomyelitis in domestic cats in South America (Bowman et al. 2008; Gómez et al. 2010; Moroni et al. 2012). The life cycle of this parasite is not fully understood, but it is thought to use snails (gastropods) as intermediate hosts, as in other metastrongyloid nematode species. Clinical symptoms of *Gurltia paralysans* infection include progressive ataxia in the hind legs, paraparesis, paraplegia, fecal or urinary incontinence, and tail paralysis. It is believed that the presence of both adult parasites and eggs together causes the appearance of clinical symptoms (Moroni et al. 2012). It has been reported that a definite diagnosis of this parasite may be made by necropsy (Gómez et al. 2010).

### Toxoplasmosis

Another endoparasite that causes lameness is *Toxoplasma gondii*. *T. gondii* is an obligate intracellular protozoan parasite that may infect almost all warm-blooded animals. While cats are the definitive host, other animals (including cats) serve as intermediate hosts (Taylor et al. 2006). It has been reported that cats may develop generalized myopathy due to *Toxoplasma*. A two-year-old female cat with kyphosis, kangaroo-like posture, and especially lameness in all four limbs, especially in the front, was observed. The application of clindamycin treatment was reported to be successful in the literature (Butts and Langley-Hobbs 2020). In our country, it has been reported that in two cats diagnosed with *Toxoplasma*, lameness in the hind legs, hyperesthesia, pain on palpation, and involuntary contractions were observed (Güven and Ceylan 2020). In another case report (Del Vecchio and Grande 2021), tetraparesis developed in an 11-month-old male cat, and the clinical signs in the patient were as follows: tremor with cerebellar ataxia, postural disorder in the front limbs and right hind limb, and *T. gondii* was confirmed as a result of necropsy. In these cases, although other causes of lameness could not be fully excluded, the presence of neurological signs alongside confirmed *T. gondii* infection—supported by necropsy in one report—suggests that toxoplasmosis was the likely underlying cause.

### Neosporosis

A case of *Neospora caninum* infection was reported in a 9-year-old female dog, initially presenting with lameness in the left hind limb, which later progressed to generalized ataxia and ultimately led to death (Galgut et al. 2010). Post-mortem examination and cerebrospinal fluid analysis confirmed the presence of *N. caninum* tachyzoites. In another case report (Knowler and Wheeler 1995), a dog infected with *N. caninum* was reported. Paraplegia in the hind legs, stiffness in the quadriceps and gracilis muscles, widespread muscle pain, muscle atrophy, and lack of perineal reflex were observed in a 15-week-old female Labrador. The patient was called for a follow-up 11 months after treatment. Although improvement was observed in the patient, muscle atrophy was observed in the hind legs, and it was reported that the patient could not walk long distances.

### Hepatozoonosis

*Hepatozoon americanum*-infected macrophages are known to localize between striated muscle fibers, forming cyst-like structures that may lead to lameness in cats and dogs (Ewing et al. 2003). It has been reported that lameness accompanied by fever, decreased tear production, mucopurulent ocular discharge, atrophy of extraocular muscles, muscle pain due to myositis, and generalized muscle atrophy is observed in most dogs diagnosed with *H. americanum* infection (Potter and Macintire 2010). It should not be forgotten that the pain associated with this disease may be localized in the lumbar and cervical spine, long bones, and joints and that walking abnormalities, limb stiffness, and even paralysis and inability to stand may be seen, and the prognosis may vary. Periosteal bone proliferation has been reported as a common finding in the radiographs of *H. americanum*-infected dogs (Panciera et al. 2000). It is also thought that this condition may cause pyogranulomatous myositis at the junctions of bone and muscle. It has been reported that even if dogs exposed to this parasite do not show myositis, there may be disturbances in the formation of long and flat bones (Panciera et al. 2000). Another study supporting these findings (Drosty et al. 2003) reported the development of hypertrophic osteopathy, showing the absence of periosteal proliferation and cortical bone destruction as radiographic and histological changes in *H. americanum*-infected patients.

### Angiostrongyliasis

*Angiostrongylus cantonensis* may lead to paralysis in the hind legs of cats and dogs. This parasite has been reported to cause lameness in 55 dogs (Mason 1987). The same study stated that the most characteristic symptoms of dogs infected with the parasite are pain during palpation and manipulation of the tail base, hind legs, and spine. Dogs were divided into three classes according to the grade of the disease, mild, moderate, and severe, and were treated with anthelmintic, corticosteroid, and supportive care. Dogs with grade 1 (mild) neurological involvement showed mild ataxia and pelvic limb paresis, which was sometimes perceived as slight hindlimb weakness or instability. Improvement was observed within 7 days, and these dogs were able to walk and run within 30 days. Some retained mild ataxia, and complete recovery was achieved by 60 days. Dogs with grade 2 (moderate) involvement displayed marked pelvic limb paresis and partial or complete loss of hindlimb function, often accompanied by lumbar hyperalgesia. Some dogs began to recover within 7–14 days, but others progressed to paralysis. In one case, the paralysis became severe and irreversible, resulting in euthanasia due to poor prognosis and inability to stand. In dogs with grade 3 (severe) disease, rapidly progressive paralysis and/or intense hyperesthesia were noted. Despite two weeks of intensive supportive care, the prognosis remained poor, and euthanasia was ultimately performed.

Another case report by Malik et al. (2013) presented a 12-week-old Golden Retriever with sudden and short-lived dysfunction in its hind legs. In addition to these symptoms, urinary and fecal incontinence and significant hyperesthesia were also reported. The diagnosis of neural angiostrongyliasis was made by examining the eosinophilic pleocytosis and antibody levels in the cerebrospinal fluid of the dog. It was reported that the dog fully recovered after symptomatic and supportive care along with corticosteroid treatment. Another study evaluating dogs infected with *A. cantonensis* reported that the most common clinical signs at the presentation were hyperesthesia, posterior proprioceptive ataxia, weakness in the hind limbs, and muscle atrophy (Lunn et al. 2012).

### Leishmaniasis

*Leishmania* spp., the protozoan parasites transmitted by sand flies, can cause systemic infections in dogs and may lead to lameness through multiple mechanisms (Morales-Yuste et al. 2022). Infected dogs not only develop clinical disease but also act as important reservoirs for zoonotic transmission to other animals and humans (Morales-Yuste et al. 2022). Clinical signs of canine leishmaniasis include weight loss, anorexia, vomiting, polyuria, polydipsia, hyperkeratosis, alopecia, interdigital inflammation, kyphosis in the dorsolumbar region, and localized pain upon palpation (Evans and Rebêlo 1996; Morales-Yuste et al. 2022). Additionally, musculoskeletal manifestations such as lameness, joint pain, and muscle atrophy have been frequently observed (Turrel and Pool 1982). Radiographic findings in infected dogs may include periosteal proliferation and increased opacity in the long bones, suggestive of inflammatory bone disease. In some cases, leishmaniasis induces immune-mediated polyarthritis, particularly Type II immune complex-mediated arthritis, which is characterized by inflammation resulting from immune complex deposition in synovial membranes (Koutinas and Koutinas 2014). This non-erosive, inflammatory joint disease has been reported in dogs with systemic infections such as leishmaniasis, bacterial endocarditis, pyoderma, pneumonia, and fungal diseases (Bennett 1987; Rondeau et al. 2005; Stull et al. 2008). These chronic infections serve as persistent sources of antigenic stimulation, promoting ongoing immune responses that contribute to joint inflammation (Koutinas and Koutinas 2014). In one case study, a young male dog presented with chronic lameness, joint swelling, and polyarthritis in the tarsal and metatarsal joints; cytological examination of synovial fluid confirmed *Leishmania* infection (Santos et al. 2006). While less studied, other parasites such as *Spirometra decipiens* have also been implicated in causing lameness, as observed in a feline case in Chile, although the mechanisms in such cases remain unclear (Fredes et al. 2022). Taken together, the pathogenesis of lameness in leishmaniasis appears multifactorial, involving direct joint involvement, immune-mediated processes, and systemic inflammatory effects.

### Tick paralysis

Tick paralysis is characterized by paralysis/limping caused directly by the tick’s own produced toxin, unlike the abovementioned ectoparasitic infestations in cats and dogs which are studied and compiled as vectors since they carry the causative agents to the host. Therefore, it is considered more appropriate to present it under a separate heading.

Ticks are blood-sucking arthropods distributed primarily in tropical and subtropical climate zones. They are classified as the second most important pests as vectors following mosquitoes (Sutherst 2004; Hornok et al. 2008). They may cause direct and indirect harmful effects on mammalians being ectoparasites and transmitting animal and human disease agents (Jongejan and Uilenberg 2004; Klompen et al. 1996). The first studies on tick paralysis were conducted by Hadwen in 1913. The results of the study revealed that tick paralysis in animals occurred about one week after contact with female ticks. Importantly, it was found that the symptoms were caused not by a disease carried by the ticks, but by a toxin produced by tick itself (Hadwen 1913).

Some tick species’ neurotoxins found in their saliva cause tick paralysis in the host they attach to. The toxins are reported to cause delays in nerve transmission and prolongation of transmission time, and symptoms start to appear on average 32 h after contact with the tick. Tick saliva, while playing an important role in the biological development of the tick, also allows for the transmission of pathogens to susceptible hosts in addition to shaping its parasitic effect (Altay and Şahin 2020). Tick species that cause paralysis vary by country (Hall-Mendelin et al. 2011; Kocoń et al. 2023; O’Neill et al. 2024). The female *Ixodes holocyclus* tick secretes a strong holocyclotoxin after attachment, which causes severe neurotoxicity in pets, including dogs, cats, cattle, sheep, horses, and goats (Masina and Broady 1999; Hall-Mendelin et al. 2011). While the exact effects of neurotoxins in ticks are not fully understood, they are thought to act similarly to *Clostridium botulinum* toxins.

While tick paralysis may initially present with mild symptoms such as lethargy, weakness, headache, and paresthesia (Felz et al. 2000), in later stages, in addition to these symptoms, muscle weakness, paralysis, and balance disorders may be observed (Laufer and Chiota-McCollum 2015). It has also been reported that in addition to damage to the extremities, paralysis of the cranial nerve may occur (Li and Turner 2004). It is emphasized that precautions should be taken against ticks in endemic areas and that a thorough tick examination should be performed in cases of acute and rapidly progressing muscle weakness, ataxia, and muscle weakness (Taraschenko and Powers 2014). A study conducted in Australia on dogs (Atwell et al. 2001) reported a significant association between individual tick counts and clinical indicators of tick paralysis, including lameness and gait abnormalities. The findings showed that lameness severity increased with higher tick burdens in individual animals, suggesting a direct relationship between parasite load and musculoskeletal impairment. The same study reported that if tick paralysis is not diagnosed and treated early, death may occur. Some ticks secrete a cement-like adhesive substance to attach themselves to their host during blood feeding. Unlike other species, *Ixodes holocyclus* directly attach to tissues with their mouths without producing a substance that anchors their mouth parts to the skin.

*Dermacentor andersoni* (Lysyk et al. 2009; Hall-Mendelin et al. 2011) is another tick species, reported to cause paralysis. *Dermacentor andersoni*, commonly known as the Rocky Mountain wood tick, affects domestic animals and is known to attach and feed more superficially compared to other tick species. This is related to the penetration of its hypostome, which cannot penetrate deep tissues (but is also supported by the secretion of an external substance) Due to this attachment/feeding method, it is thought to cause less severe paralysis compared to the *Ixodes holocyclus* tick (Hall-Mendelin et al. 2011). Therefore, its removal may result in a faster recovery.

*Rhipicephalus sanguineus* is one of the reason for paralysis in dogs. Otranto et al. (2012) reported that 14 dogs infested with *Rhipicephalus sanguineus* ticks showed varying degrees of neurological symptoms, including hindlimb incoordination, lameness, generalized weakness, and difficulty in movement. All dogs were heavily infested, with tick counts ranging from 63 to 328 per animal. Despite treatment, 10 of the dogs died within 24 h of presentation, while the remaining four recovered within three days after administration of a spot-on formulation containing Fipronil and (S)-Methoprene (Otranto et al. 2012). Progressive ataxia was evaluated in a 9-year-old miniature horse. Clinical findings observed in the patient were reported to be a weakness in the hind limbs, ataxia, and dysfunction of lower motor neurons, and *Ixodes holocyclus* ticks were detected on the patient’s upper (head and neck) part (Tee and Feary 2012).

## Parasites and lameness in ruminants

**Fig. 2:**
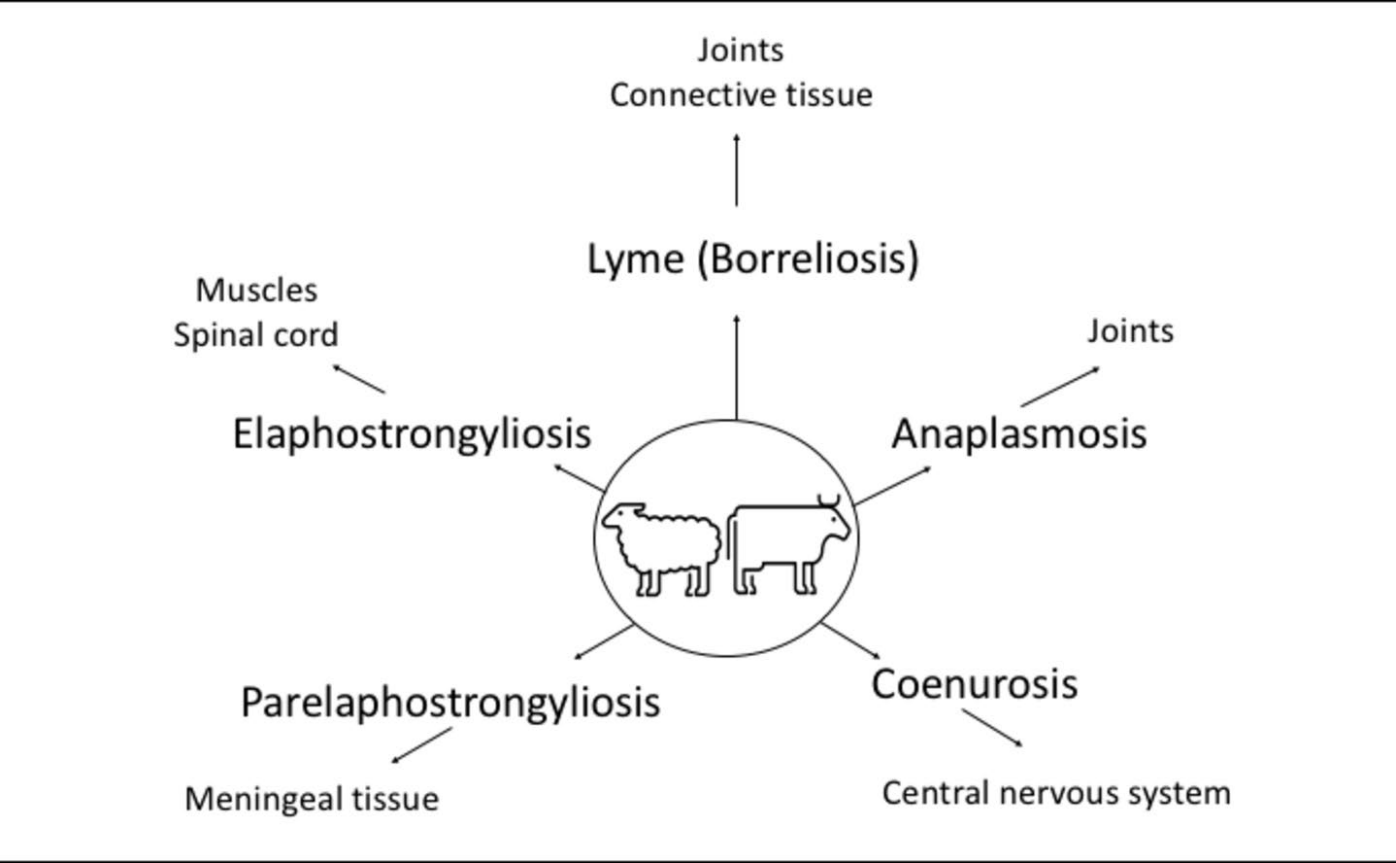
Parasitic diseases and affected structures and tissues in ruminants

Parasitic diseases and affected structures and tissues in ruminants presented in Fig. 2. Information regarding each disease are presented under separate headings in the following sections.

### Tick infestations

Tick infestations in ruminants may lead to anemia, loss of productivity, and welfare problems. In addition, they may cause myiasis, abscesses, lameness, neurological symptoms, and even fatal cases. It has been demonstrated that ticks tend to prefer certain body regions of domestic ruminants for attachment and blood feeding (Nasirian 2022). In goats, the tail and anal region, ear region, and udder or scrotum are among the most frequently infested areas, followed by the interdigital space of foot, which is also commonly affected (Jongejan et al. 2020; Nasirian 2022). In a study, it was reported that adult and nymph *Amblyomma hebraeum* ticks prefer to attach to the interdigital area of goats’ feet, which frequently leads to secondary infections and lameness (Jongejan et al. 2020). The formation of foot abscesses in goats has been reported to be associated with the seasonal abundance of adult *Amblyomma hebraeum* and *Rhipicephalus glabroscutatus* ticks (Jongejan et al. 2020). Furthermore, certain tick species in different ruminants, such as *A. hebraeum* and *R. glabroscutatum* may cause lameness in goats, while *Hyalomma rufipes* is responsible for the same condition in sheep (Onyiche and MacLeod 2023).

In a study on lameness in Merino sheep and lambs (Kok and Fourie 1995), the presence of *Hyalomma rufipes* and *Hyalomma truncatum* ticks was highlighted. It was found that lambs were affected more frequently and more severely than sheep, as they exhibited higher tick burdens and more consistent signs of lameness, particularly associated with tick attachment to the feet and lower legs.

In another study (Azizi and Yakhchali 2006), lameness observed in a flock of Makui breed sheep was investigated. A high population of ticks localized on the legs of the sheep (invading their legs) was observed in this flock. The lameness rate was found to be higher in lambs (17%) than in adults (3.5%), and it was reported that lameness was associated with the presence of two or more ticks in the femoral region of lambs. The ticks encountered in the study were mostly *Hyalomma anatolicum* and *Hyalomma asiaticum* species. They suggested that ticks caused lameness by causing tissue damage and inflammatory reaction.

In a goat farm in India (Soundararajan 2019), 65 Osmanabadi goats were examined for lameness. Five of these goats showed clinical signs, and in two young females, tick infestation (*Hyalomma isaaci*) was identified in the interdigital space and on the lower limbs. These ticks were associated with dried blood clots, serous fluid, and necrotic tissue at the bite sites. The study concluded that tick infestation should be considered a potential cause of lameness in goats.

### Lyme (Borreliosis)

Arthritis and related lameness were observed in ruminants infected with the tick carrying the causative agent of Lyme disease, *Borrelia burgdorferi* (Eze 2002). The presence of spirochetes in serum and joint fluid samples was reported to be helpful in reaching this diagnosis. However, it has also been reported that ticks and ectoparasitic mites (e.g., *Sarcoptes* spp.) affecting the feet can contribute to lameness in ruminants (Eze 2002).

### Coenurosis

There are also endoparasite species that can lead to lameness in ruminants. *Coenurus cerebralis*, the larval form of *Taenia multiceps*, localizes in the central nervous system, particularly the brain, of sheep, goats, cattle, horses, and humans (Loos-Frank 2000; Scala and Varcasia 2006; Doğanay and Vural 2012; Varcasia et al. 2022). The presence of *C. cerebralis* in the nervous system of sheep and other ruminants—including the brain and spinal cord—has been documented (Scala and Varcasia 2006; Sharma and Chauhan 2006; Scala et al. 2007; Batista et al. 2010; Varcasia et al. 2022). Additionally, the parasite has occasionally been identified in other organs, subcutaneous tissues, and muscle (Sharma and Chauhan 2006; Sharma et al. 2021; Varcasia et al. 2022). Due to the pressure exerted on the brain by *C. cerebralis*, neurological signs such as circling, bruxism (teeth grinding), loss of balance, and torticollis (neck twisting) may be observed. In severe cases, cerebral atrophy, softening and thinning of the skull bones, and eventual death can occur (Varcasia et al. 2022). Moreover, nodular lesions of varying sizes have been described in different tissues of goats (e.g., subcutaneous and muscular tissues), which can result in lameness (El Sinnary et al. 1999; Sharma et al. 2021).

*Taenia gaigeri*, considered a larval form of *Taenia multiceps* similar to *Coenurus cerebralis*, forms cyst-like structures not in the central nervous system, as typically observed in sheep and goats, but rather in other tissues such as the subcutaneous fascia and intramuscular regions (Jackson 1919; Bhalla and Nagi 1962; Singh and Singh 1972; Oryan et al. 2012; Varcasia et al. 2012; Sharma et al. 2021). When these cysts develop in locomotor-associated tissues, they can cause lameness by inducing pain, tissue degeneration, necrosis, muscle atrophy, or secondary infections, all of which contribute to impaired muscle function.

### Parelaphostrongyliosis

Abnormal migration of *Parelaphostrongylus tenuis*, which is known as the normal host of white-tailed deer (*Odocoileus virginianus*), may also causes lameness, paralysis, and neurological symptoms in ruminants (Rickard et al. 1994). It has been reported that this may lead to lameness in the host due to the damage caused by the parasite’s abnormal migration to the spinal parenchyma and the resulting inflammatory response. The onset of clinical symptoms has been reported to occur 4 to 8 weeks after infection (Rickard et al. 1994). The first signs of this condition have been emphasized to be a short, stiff gait with dragging of the hind limbs. Similar findings have also been diagnosed in the front limbs in the later stages. In an experimental study conducted with the same parasite (Pybus et al. 1996), ataxia, paresis, weakness, and ultimately inability to stand were described in infected sheep. In experimental studies, the severity of clinical signs in infected animals was observed to increase with higher larval loads, and the presence of *P. tenuis* larvae was confirmed through the detection of first-stage larvae in fecal samples using a modified Baermann technique, as well as through necropsy and histopathological examination of the central nervous system (Pybus et al. 1996). Naturally infected cattle, sheep, goats, and llamas were defined to show paralysis, unilateral and/or bilateral hindlimb lameness, and weakness as the first clinical signs in infected animals (Jortner et al. 1985; Pybus et al. 1992; Duncan and Patton 1998). New reports continue to be published on newly discovered species, sitatunga, infected with *P. tenuis* (Diaz-Delgado et al. 2021). Publishing more case reports would enhance understanding of parasites’ effects on lameness.

### Elaphostrongyliosis

Another nematode species that may cause lameness is *Elaphostrongylus cervi*. It has been reported that adult worms live in the connective tissue within the muscles and under the epimysium of skeletal muscles of infected animals, while mature parasites and larvae in their third or fourth stage may be found in the subdural and subarachnoid spaces of the central nervous system (Kutzer and Prosl 1975). In a study conducted by Alberti et al. (2011), fecal samples were collected from 31 red deer, five goats, and one sheep. The small ruminants had shown neurological symptoms, and copper deficiency was diagnosed in one goat. Spinal ataxia and asymmetric paraparesis were observed in sheep and goats, with some attempting to walk on their carpal joints. Histopathological examination revealed severe neurological lesions in five of the six animals (one sheep and four goats). PCR confirmed infection with *E. cervi* in two of the goats (Alberti et al. 2011). In another study in Norway involving 13 goats showing neurological symptoms (Handeland and Sparboe 1991), a necropsy of the goats revealed *E. rangiferi* nematode and its eggs in the central nervous system of nine of the goats. Patients’ clinical symptoms were reported to include itching, motor weakness, paralysis, lameness, decreased vision, circling, abnormal head position, and scoliosis, which were likely due to damage caused by mature or nearly mature nematodes. Various parasites and hosts associated with lameness in ruminants are summarized in Table 2.

**Table 2:** Parasitic diseases associated with lameness in ruminants: causative agents, intermediate hosts, tissue involvement, and diagnostic methods

Diseases	Causative		Tissue	Diagnosis method
	Intermediate host(s)	Parasite(s)		
Lyme (Borreliosis)	*Ixodes ricinus*	*Borrelia burgdorferi*	Joint Connective Tissue	PCR
Anaplasmosis	*Ixodidae* spp.	*Anaplasma phagocytophilum*	Joint	Synovial Fluid Analysis
Coenurosis	*Taenia multiceps*	*Coenurus cerebralis*	CNS	Necropsy
Parelaphostrongyliosis	Gastropods	*Parelaphostrongylus tenuis*	Meningeal Tissue	Necropsy PCR
Elaphostrongyliosis		*Elaphostrongylus cervi*	Muscle Spinal Cord	Necropsy PCR

*PCR* Polymerase chain reaction, *CNS* Central nervous system

## Parasites and lameness in equids

**Fig. 3:**
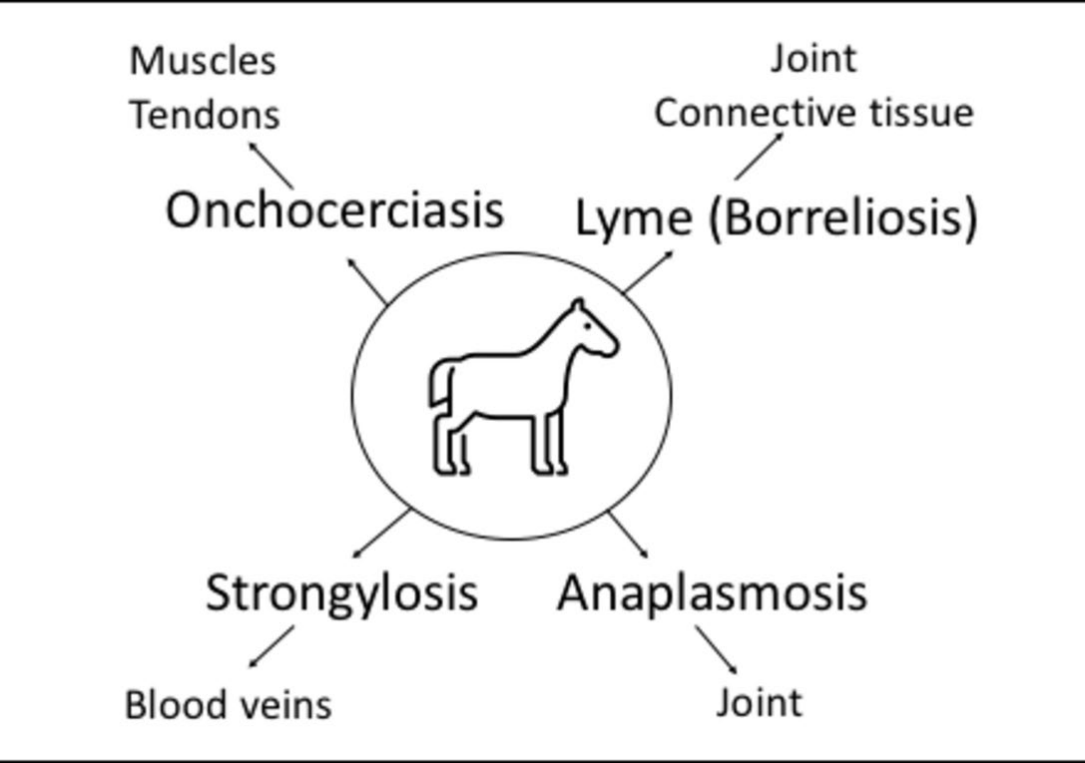
Parasitic diseases and affected structures and tissues in equids

Parasitic diseases and affected structures and tissues in equids presented in Fig. 3. Comprehensive details regarding each are provided under distinct headings in the sections that follow.

### Lyme (Borreliosis)

Ticks serve as vectors in equids as in other mentioned animals. *Borrelia burgdorferi* sensu lato, a spirochete transmitted by ticks of the *Ixodes* spp., is the causative agent of Lyme borreliosis. Clinical signs of Lyme borreliosis in horses include chronic weight loss, sporadic lameness, arthritis, laminitis, low-grade fever, swollen joints, muscle soreness, and uveitis. In chronic cases, neurological signs such as depression, behavioral changes, difficulty swallowing, head tilt, and encephalitis may also be observed (Parker and White 1992; Egenvall et al. 2001; Butler et al. 2005).

In a study conducted on ponies experimentally infected with *B. burgdorferi*, various tissue samples were collected, including skin biopsies taken at monthly intervals from the site of tick exposure. Postmortem tissues, including skin, fascia, skeletal muscles, synovial membranes, lymph nodes, joint capsules, myocardium, pericardium, kidneys, and other organs, were also obtained and tested for the presence of *B. burgdorferi* using culture and PCR techniques. Positive results were reported in culture samples taken from these tissues. *B. burgdorferi* was most frequently isolated from the skin, fascia, and muscles of the ponies, however no clinically significant symptoms were observed in any of them. Microscopic lesions were limited to the skin and peripheral lymph nodes near the sites where the ticks were attached in most ponies, while some showed mild, non-suppurative synovitis, perineuritis, and meningitis (Chang et al. 2000). A study conducted in Italy (Ebani et al. 2015) to determine the incidence of tick-borne infections concluded that although the clinical incidence of borreliosis is rare in the equine population, the observed prevalence indicates that *B. burgdorferi* may affect horses. Therefore, *B. burgdorferi* infection should be considered in the differential diagnosis, especially in cases of lameness (Ebani et al. 2015). Another reported case study showed that *B. burgdorferi* caused polysynovitis in horses. Clinical symptoms observed in the horse included fever, intermittent lameness, and effusion of the digital flexor tendon sheath in the right hind limb. A definite diagnosis of polysynovitis caused by *B. burgdorferi* sensu lato. infection was made based on serological tests (Passamonti et al. 2015). However, the relationship between *B. burgdorferi* infection in horses and stiffness and lameness was not documented, and there was no evidence that the infection caused laminitis (Divers et al. 2018).

### Anaplasmosis

Another factor that causes lameness, equine granulocytic anaplasmosis, is caused by *Anaplasma phagocytophilum*, an obligate intracellular bacterium transmitted by ticks. The vector in the transmission of this disease is ticks of the *Ixodes* genus; the main vectors in Europe are *Ixodes ricinus*, and in North America, *Ixodes pacificus* and *Ixodes scapularis* (Dzięgiel et al. 2013). Studies have shown that at the onset of infection, the horse exhibits atypical symptoms, making it difficult to diagnose. Later, fever, apathy, partial anorexia, stiff gait, reluctance to move, painful swelling in the fetlock joint, and eventually lameness develop in the horse (Giudice et al. 2011; Saleem et al. 2018). In a study of 50 horses admitted to a clinic, 54% of the horses were found to have ectoparasites (ticks), and 58% were infected with endoparasites, with the most common endoparasite being the *Strongylidae* spp. nematode. Pathological changes such as tenosynovitis, tendinitis, increased joint volume, and lameness were reported in 46% of the evaluated animals (Soares de Andrade et al. 2009). However, the relationship between parasites and lameness has not been investigated.

### Onchocerciasis

Endoparasitic infestations have a negative impact on the life of ungulates, and it is believed that some parasites that settle in the muscles and tendons of racehorses cause lameness. The most well-known of these parasites are nematodes of the *Onchocerca* genus. *Onchocerca* includes a nematode species that lives in different anatomical regions of the subcutaneous tissues, ligaments, and aponeuroses of domestic animals and may form more than thirty nodules (Anderson 1999). Infection with *Onchocerca reticulata* is characterized by swelling and lameness in the extremities, usually with the presence of subcutaneous nodules over or within the flexor tendons and suspensory ligaments (Anderson 1999). In a study conducted in Italy, swelling was observed in the right metacarpal region of a horse brought to the hospital with suspected tendinitis. According to the anamnesis, this lesion had occurred six months earlier and had gradually increased in size. During the clinical examination, a hard and painless mass located palmaro-laterally in the proximal third of the right metacarpal region and a slight swelling in the medial direction of the left metacarpal region were observed, along with mild lameness in the horse. The presence of a mass and parasite was confirmed by ultrasound. Histopathological examination of the sample taken from the area confirmed the diagnosis of *Onchocerca boehmi* filariasis (Lia et al. 2017). In another study by Paraschou et al. (2021), three donkeys were brought to the hospital with complaints of progressive lameness. Chronic lameness was observed in the front legs of the first case, the hind legs of the second case, and all four legs of the third case. Due to the worsening condition of the patients, euthanasia was performed, and *Onchocerca* spp., which causes degenerative suspensory ligament desmitis was diagnosed (Paraschou et al. 2021).

### Strongylosis

Another parasite that manifests symptoms of lameness is *Strongylus vulgaris*. It has been reported that this parasite causes aortic iliac thrombosis (Oyamada et al. 2007). In the reported case, intermittent lameness that gradually increased was observed in two horses. Macroscopically, thrombi were detected in the abdominal aorta caudal to the kidneys and in many of its branches in both horses. Emboli in the femoral artery and narrowing of the vessel lumen was also reported. Various parasites and hosts associated with lameness in equids are summarized in Table 3.

**Table 3:** Parasitic diseases associated with lameness in equids: causative agents, intermediate hosts, tissue involvement, and diagnostic methods

Diseases	Causative		Tissue	Diagnosis method
	Intermediate host(s)	Parasite(s)		
*Lyme (Borreliosis)*	*Ixodes genus*	*Borrelia burgdorferi*	Joint Connective Tissue	PCR
*Anaplasmosis*	Ixodidae spp.	*Anaplasma phagocytophilum*	Joint	Synovial Fluid Analysis
*Onchocerciasis*	*Simulium yahense*	*Onchocerca reticulate* *Onchocerca boehmi*	Muscles and Tendons	Histopathological Examination USG
*Strongylosis*	Infected Feces	*Strongylus vulgaris*	Blood Veins	Fecal Culture

*PCR* Polymerase chain reaction, *USG* Ultrasonography

## Parasites and lameness in pigs

**Fig. 4:**
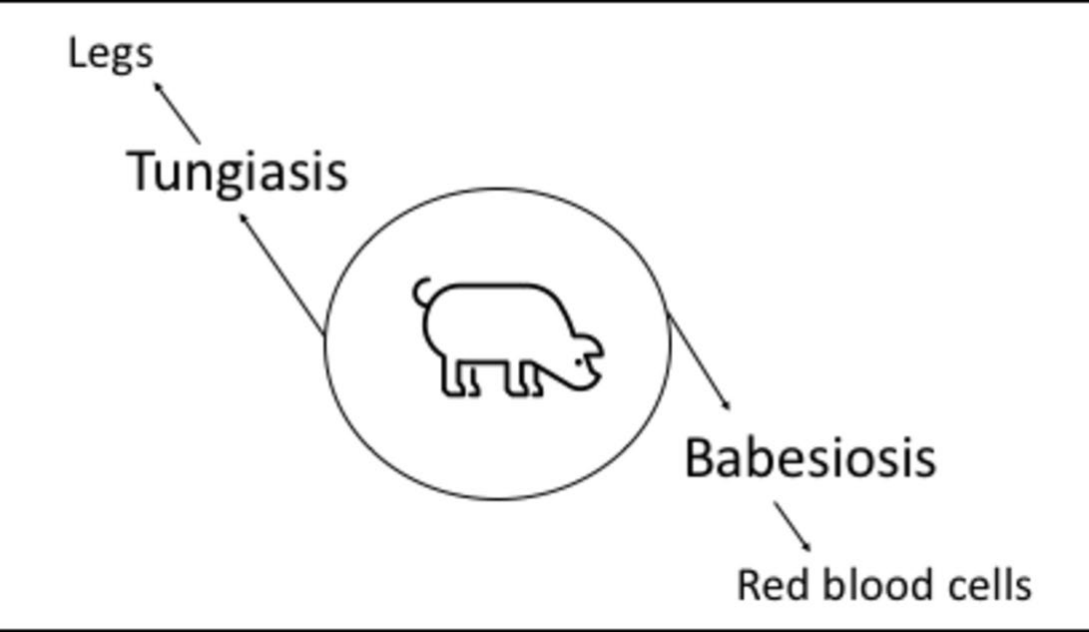
Parasitic diseases and affected structures and tissues in pigs

Figure 4 presents the structures and tissues affected by parasitic diseases in pigs. Further information is provided in the sections below.

### Tungiasis

Skin-embedded female sand fleas (*Tunga penetrans* and *Tunga trimamillata*) cause a zoonotic disease called tungiasis. Heukelbach et al. (2004) and Mutebi et al. (2016) reported that the lesions caused by these fleas in pigs (such as nail cracks, hyperkeratosis, alopecia, and edema) are mostly localized in the limbs, causing lameness.

### Babesiosis

The other parasitic factor associated with lameness is Babesiosis, which is commonly observed in farm animals but less frequently in pigs. Zobba et al. (2020) reported clinical signs such as anorexia, depression, high fever, reluctance to move, and lameness in pigs. Various parasites and hosts associated with lameness in pigs are summarized in Table 4.

**Table 4:** Parasitic diseases associated with lameness in pigs: causative agents, intermediate hosts, tissue involvement, and diagnostic methods

Diseases	Causative		Tissue	Diagnosis method
	Intermediate host(s)	Parasite(s)		
Tungiasis	Female Sand Flea	*Tunga penetrans*	Legs	PE, Clinical Findings
Babesiosis	*Rhipicephalus sanguineus*	*Babesia trautmanni* *Babesia perroncitoi*	RBC	Cytological Examination Necropsy

*RBC* Red blood cells, *PE* Physical examination

## Conclusion

**Fig. 5:**
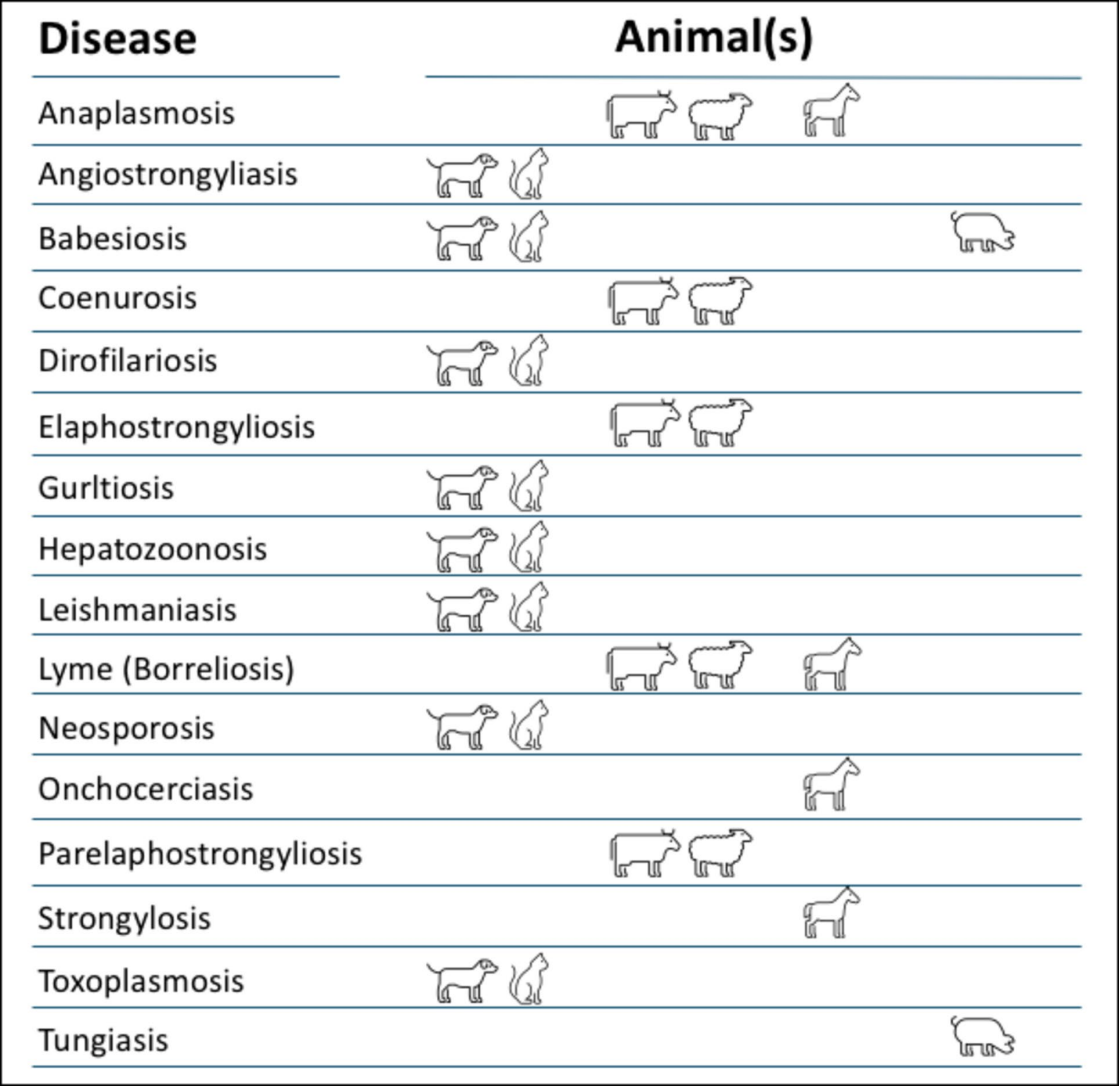
Parasitic diseases associated with lameness and affected animal species

This review aims to address the issue of parasite-induced lameness in animal species and generate interest in potential cases. A summary of parasitic diseases associated with lameness in animal species is presented in Fig. 5.

One of the limitations of this review is the disproportionate emphasis on the epidemiological features, biological cycles, clinical signs, and diagnostic findings of parasitic diseases associated with lameness, largely derived from available case reports. In several instances, limited data were available regarding the specific pathogenesis leading to lameness, the extent of lesions, and their reversibility. As such, information concerning treatment efficacy, prognosis, long-term outcomes, and implications for animal productivity, welfare, and quality of life remains insufficient. Future studies should aim to address whether the lameness observed in parasitic infections is reversible with proper treatment or represents permanent musculoskeletal or neurological damage, as this has important practical implications for veterinarians, animal owners, and farmers.

In conclusion, lameness is a common presenting symptom in domestic animals. While routine clinical examinations may help to identify the underlying cause of lameness, there may be cases where a definitive diagnosis cannot be established. Parasitic infections could result in various clinical appearances, including musculoskeletal disorders that may present as lameness and be overlooked during routine clinical evaluations. Therefore, it is crucial to consider the possibility of parasitic infections in cases of undiagnosed lameness in domestic animals. A thorough consultation with a parasitologist may be required to achieve a correct diagnosis. This will enable timely and proper management of the underlying condition, improving animal welfare and reducing economic losses associated with undiagnosed and untreated parasitic infections. In lameness cases in which a final diagnosis may not be reached, parasitic examination should be taken into consideration. Thus, conducting a consultation with a parasitologist when necessary may be an invaluable step in the diagnostic work-up of domestic animals, ensuring appropriate and effective management of the animal’s condition.

## Data Availability

No datasets were generated or analysed during the current study.
